# The perceptions of medical students of online teaching during the COVID-19 pandemic: a national survey from Jordan

**DOI:** 10.25122/jml-2023-0116

**Published:** 2024-04

**Authors:** Montaha AL-Iede, Jumana Albaramki, Ayah Alsoudi, Ruqaya Al-Ani, Faiha`a AL-Akhras, Rana Al Najada, Sondos Al-Najjar, Rawan AL-Sallal, Al-Motasem Yousef, Shereen Aleidi, Basim Alqutawneh

**Affiliations:** 1 Department of Pediatrics, Jordan University Hospital, Amman, Jordan; 2 School of Medicine, The University of Jordan, Amman, Jordan; 3 Department of Biopharmaceutics and Clinical Pharmacy, School of Pharmacy, The University of Jordan, Amman, Jordan; 4 Department of Radiology, Blacktown/Mount-Druitt Hospital, Sydney, Australia

**Keywords:** medical education, COVID-19, challenges

## Abstract

In response to the COVID-19 pandemic, Jordan declared a state of emergency on 19 March 2020, implementing a 10-week curfew and closing all educational institutions. Consequently, online learning commenced to ensure educational continuity amid the pandemic. The aim of this study was to assess medical students’ perception of online teaching during this period in a limited-resource setting and to identify associated challenges. A cross-sectional survey was conducted involving 393 undergraduate medical students in their 4^th^, 5^th^, and 6^th^ year from six universities across Jordan. The self-administered online survey included four categories, exploring the satisfaction of medical students and challenges they faced during online education, and was distributed on Facebook and WhatsApp. A total of 393 students completed the survey, 264 (62.6%) of which were female. The majority of respondents were from Jordan University and in their 4^th^ year. Regarding online teaching, 218 (55.5%) expressed satisfaction; however, an equivalent percentage disagreed that online methods could replace traditional teaching. Notably, 238 (86%) believed that their confidence in new clinical skills acquired through online education was adversely affected. The study highlights the need for targeted interventions to improve the effectiveness of online education, especially in developing essential clinical skills.

## INTRODUCTION

The world has been profoundly impacted by COVID-19, a contagious respiratory infection caused by the SARS-CoV-2 virus [1]. The outbreak was declared a pandemic by the World Health Organization on 11 March 2020 [2]. Because of the COVID-19 pandemic, several universities and colleges have transitioned from face-to-face teaching methods to online teaching or blended teaching, a combination of the two methods [3].

In Jordan, the first diagnosed case of COVID-19 was reported in early March 2020 [4]. Since then, there has been a steady increase in the incidence of the disease, with a total of 1.18 million confirmed cases and 13,142 deaths in the country by 28 January 2022 [5]. A curfew was declared on 21 March 2020, and the implemented lockdown restrictions have affected all aspects of life. Medical education has also been affected, with colleges and universities shut down. Educational activities, such as lectures, clinical sessions, and examinations, were suspended, leading to a sudden shift toward online education. This transition enabled students to continue their education remotely during the COVID-19 pandemic.

The medical education program in Jordan spans six years, with the last three years dedicated to clinical training. In the first three years of the program, students receive in-class theoretical education through lectures, seminars, and in-hospital clinical rotations. Online education was not used as a modality for medical teaching before the COVID-19 pandemic.

Online education may represent a suitable alternative to traditional education methods for delivering a good-quality medical education. However, important challenges include the need for essential infrastructure and efficient institutional strategies [6]. The issues reported with online education include difficulties with time management, lack of interaction, and student evaluation [7]. Additionally, the quality of teaching and the availability of equipment required for online education also pose challenges, as some students do not have access to computers and high-speed internet [8]. Moreover, many teachers struggle with using computer software and hardware or troubleshooting systems during online classes [9].

For medical students, losing connectedness with their peers and the community has been particularly challenging. The clinical practice years of medical education include case-based learning sessions, clinical rounds, attending clinics, and participating in surgical operations within hospitals [10,11]. Replacing this hands-on experience with online learning has been difficult for medical students, raising doubts about the effectiveness of online learning for the development of clinical skills compared to traditional education. Students have also expressed concerns about exam submissions, objective structured clinical examination (OSCE) stations, and the scheduling of electives [10,11].

On the other hand, online education has been reported to have a positive effect, leading to broad acceptance of technology-enabled education [12]. Advocates of this positive view believe that online teaching is as effective as face-to-face classroom education [13]. Transitioning to online education required significant support from the university and the provision of necessary tools [14]. In September 2020, the government announced the return of clinical year students to hospital training, with the requirement to use personal protective equipment (PPE) and adhere to social distancing guidelines. Medical students have been conflicted about returning to in-person education. Although they recognized the benefits of developing their clinical skills and communicating with the community, they also feared the possibility of contracting the virus and spreading it to their families [15].

The aim of this study was to explore the distance e-learning situation among Jordanian medical students to identify possible limitations, challenges, student satisfaction, and perspectives for online learning during the pandemic. We aimed to address the lack of comprehensive studies on the specific challenges and perspectives of Jordanian medical students amidst the transition to online education during the COVID-19 pandemic.

## METHODS

### Study design and data collection

We conducted a questionnaire-based descriptive cross-sectional study involving five medical schools from five different cities in Jordan: Jordan University, Hashemite University, Jordan University of Science and Technology, Mut’ah University, and Al-Yarmouk University. The study population was undergraduate students in their clinical practice years (4^th^, 5^th^, and 6^th^ years). Data collection occurred from 17 to 24 November 2020 using an online questionnaire designed with Google Forms and distributed through Facebook and WhatsApp. A total of 393 students from the six medical schools filled out the questionnaire. For the purposes of this study, online learning was defined as education conducted over the internet without real-life meetings of the participants.

### The questionnaire

The online questionnaire was developed based on online learning experiences reported in the literature before and during the COVID-19 pandemic. The internal validity and accuracy of the questionnaire was assessed with multidisciplinary input. A pilot study was conducted involving seven medical students, and modifications were made according to their feedback. The self-administered questionnaire contained a section informing study participants about the objectives of the study, and that the information they provide would be used solely for study purposes. The questionnaire was written in English and featured two types of questions: multiple-choice and three-response questions (agree, neutral/I do not know, and disagree) using a modified Likert scale.

The questionnaire consisted of 31 questions divided into four categories. The first category gathered sociodemographic information (age, sex, academic year, residence, university, and history of medical illnesses). The second category included questions related to online learning, addressing the timing of lectures, preparedness of the teaching staff, seminar preparation, and availability of feedback. The third category included questions about the availability of devices and tools needed for online learning, technical problems, internet connection issues, and whether the environment was comfortable for online sessions. Questions in the last category explored students’ experiences and perceptions of online learning, including their satisfaction, the perceived effectiveness and fairness of the learning method, and their confidence in the clinical skills learned online.

The survey was distributed via social media platforms such as Facebook and WhatsApp, ensuring the privacy and confidentiality of the collected data. Data exposure was limited to research members only, and the questionnaire was filled out anonymously by medical students.

### Statistical analysis

Statistical analyses were performed using SPSS v.25.0 (IBM Corp). Continuous variables are presented as mean ± s.d. or median (range) depending on normality, and categorical variables are presented as frequency and percentages. The analysis included descriptive statistics and the chi-squared test. A *P* value of ≤0.05 was considered statistically significant.

## RESULTS

Sociodemographic characteristics of the participants

The total number of undergraduate medical students who participated in the study was 393, of which 246 (62.6%) were women. The students were divided into three groups by age, and only 1% were older than 25 years. Most of the participants, 60.6% (*n* = 238), lived in the capital, Amman. More than a third of the students were in their last year, and only 4.8% (*n* = 19) got infected with SARS-Cov-2 (Table 1).

**Table 1:** Demographic characteristics of participants

Variables	*n*	(%)
**Sex**		
Female	246	62.6
Male	147	37.4
**Age, years**		
20–22	192	48.9
23–25	197	50.1
26–28	4	1
**Place of living**		
Amman	238	60.6
North of Jordan	135	34.35
South of Jordan	20	5
**University**		
Jordan University	189	48
Jordan University of Science and Technology	74	18.8
Hashemite University	64	16.3
Mut’ah University	43	10.9
Al-Yarmouk University	23	5.8
**Academic Year**		
4^th^	127	32.3
5^th^	93	23.7
6^th^	173	44
**Infected with COVID-19**		
Yes	19	4.8

### The opinions of medical students about tool availability and the online teaching environment

Participants reported minimal participation in online teaching, with only 32 (8.1%) of the students being often active during online classes. However, most students indicated that electronic devices were always available, and that their home environment was comfortable. Technical problems and internet connection issues were rarely encountered during online teaching (Table 2). The majority of participating students, 199 (50.6%), admitted to being sometimes distracted and involved with other activities during online classes. Regarding the preparedness of tutors for online sessions, 247 (63%) of the students felt that the instructors were sometimes prepared, whereas only 67 (17%) reported that the instructors were well-prepared and effectively used multimedia to achieve lesson objectives (Table 2).

**Table 2:** Assessment of processes, tools, internet connection, and environment of online teaching (n = 393)

Statement	Always/Often	Sometimes	Rarely
Active participation during the online session	32 (8.1%)	109 (27.7%)	252 (64.1%)
Availability of devices (laptop, PC, etc.)	285 (72.5%)	96 (24.4%)	12 (3.1%)
Comfortable environment	249 (63.4%)	98 (24.9%)	46 (11.7%)
Tool-related technical issues	35(8.9%)	126 (32.1%)	232 (59%)
Internet connection issues	53 (13.5%)	152 (38.7%)	188 (47.8%)
Teacher’s preparedness for online lessons	67 (17%)	247 (62.9%)	79 (20.1%)
Involvement with other activities during an online lesson	115 (29.3%)	199 (50.6%)	79 (20.1%)

### Participants’ perceptions of online education

Almost half of the participants were satisfied with online teaching. However, only 24% agreed that online education was effective for medical students in their clinical practice years, and almost 80% believed that online examinations were unfair. In addition, 221 students (56.3%) considered that online education cannot replace traditional education in terms of delivering knowledge. Furthermore, almost half (45%) of the participants stated that distance e-learning was less effective in teaching new clinical skills, and the majority (70.7%) did not feel confident regarding the clinical skills learned through online classes (Table 3). Despite these challenges, some students reported positive aspects of online education, such as better time management (40%) and having valuable recorded material for future studying (51%).

**Table 3:** Students’ perception on online teaching (level of agreement)

Statement	Agree	Neutral/I don’t know	Disagree
I am satisfied with online teaching	218 55.5%)	92 (23.4%)	83 (21.1%)
The learning method is effective and fair	95 (24.2%)	98 (24.9%)	196 (49.9%)
Online teaching can replace traditional teaching	96 (24.4%)	73 (18.6%)	221 (56.3%)
Recorded online lectures are helpful for studies	205 (50.9%)	122 (31%)	66 (17%)
Time management is better compared to the traditional method	170 (43.3%)	65 (16.5%)	158 (40.2%)
Online teaching is effective in learning new clinical skills	28 (7.1%)	188 (47.8%)	177 (45%)
I am confident regarding the acquired clinical skills	58 (14.8%)	57 (14.5%)	278 (70.7%)
Online examinations are fair	69 (17.9%)	10 (2.5%)	314 (79.9%)
If the pandemic continues, I expect online teaching to be more effective than the traditional method	218 (55.4%)	92(23.4%)	83(21.1%)

The perceptions of 4^th^- and 5^th^-year medical students regarding the effectiveness of online teaching and the acquisition of new clinical skills were similar to those of 6th-year students, with no statistically significant difference (Table 4).

**Table 4:** Perception of medical students in their last year compared to those in the 4^th^ and 5^th^ years

Statement	6^th^ year (*n* = 175)	4^th^ and 5^th^ year (*n* = 218)	*P* value
**Online teaching is effective in learning new clinical skills**.			
Agree	14 (8.0%)	14 (6.4%)	
Neutral	85 (48.6%)	103 (47.2%)	0.5
Disagree	76 (43.4%)	101 (46.3%)	
**I am confident regarding the acquired clinical skills**.			
Agree	27 (15.3%)	31 (14.2%)	0.35
Neutral	22 (12.6%)	35 (16.1%)	
Disagree	126 (72%)	152 (69.7%)	
**If the pandemic continues, I expect online teaching to be more effective than the traditional method**.			
Agree	79 (45.7%)	139 (63.2%)	0.003^*^
Neutral	57 (32.6%)	35 (16.1%)	
Disagree	39 (22.5%)	44 (18.6%)	

* Statistically significant

Students from universities outside the capital reported internet connection issues, technical problems, and a lack of laptops compared to students studying at Jordan University. However, the differences were not statistically significant (*P* = 0.07, *P* = 0.12, and *P* = 0.44, respectively) (Table 5).

**Table 5:** Comparison between Jordan University and universities in rural areas in terms of online tools availability

Variable	Jordan University	Other universities outside Amman	P value
**Internet connection issues during online teaching/examinations**			
Always/often	20 (11.2%)	33 (15.9%)	
Sometimes	66 (36.9%)	86 (41.5%)	0.07
Rarely	93 (52%)	88 (42.5%)	
**Issues with technology tools during online teaching**.			
Always/Often	11 (6.1%0	18 (8.7%)	0.12
Sometimes	50 (27.9%)	76 (36.5%)	
Rarely	118 (56.9%)	114 (54.7%)	
**Availability of laptops/tablets/PCs to attend online teaching**			
Always	134 (74.9%)	145 (69.7%)	0.44
Most of the time	41 (22.9%)	55 (26.4%)	
Rarely	4 (2.2%)	8 (3.8%)	

## DISCUSSION

This study aimed to evaluate medical students’ experiences with computer-mediated distance e-learning, a newly adopted approach in Jordanian medical schools. Distance e-learning emerged as a new teaching method to maintain the continuity of medical education during the COVID-19 pandemic. This study assessed students’ opinions on the challenges and limitations they faced during this new learning experience, the performance of faculty staff, their overall satisfaction, and future perspectives. Traditional face-to-face teaching has long been a fundamental approach in medical education [16]. However, as clinical demands increase and available time decreases [17], a shift towards online, distance, or electronic learning has been observed in the last few decades [18]. Implementing distance e-learning in medical education is challenging, especially in low-middle-income countries.

The lack of infrastructure, technology, and internet access are barriers that affect both learners and faculty members [7], as demonstrated by the challenges reported by our study participants (Table 2). A study by Aljaraideh *et al*. identified deficiencies in the online learning infrastructure as a major barrier preventing students in Jordan from effectively using online learning [19]. Our participants also reported difficulties in maintaining attention to the educational material and becoming involved in other activities during online sessions. Similarly, a study conducted at a medical college in Nepal explored the challenges encountered by educators with e-learning, such as loss of learner attention. They found that educators used methods like interactive question-answer sessions, quizzes, and assigning specific topics for student presentations to overcome these issues [10,20].

Our study participants reported internet connection issues, particularly those from universities outside the capital city, Amman. Al-Balas *et al*. investigated the adoption of distance e-learning among medical students in Jordan during COVID-19 and found a 26.8% overall satisfaction rate, with internet streaming quality being an important challenge for 69.1% of students [4]. Muflih *et al*. also reported high perceived barriers, notably unstable internet connections and lack of motivation among medical students [21]. Atreya *et al*. highlighted additional barriers to online education, faced by both educators and learners, including difficulty finding a quiet place owing to quarantined families, interrupted internet connections and electricity due to heavy rain and hailstorms, and lack of internet access for students from poorer families [10].

E-learning has been reported to provide adequate access to a vast amount of diverse information [22]. Rajab *et al*. noted a positive impact of the COVID-19 pandemic on online medical education, with most of the participants reporting increased confidence and a desire to integrate the online expertise gained during the pandemic into their practice [3]. However, the biggest challenges faced by respondents included issues related to communication, student assessment, use of technology tools, online experience, time management, as well as pandemic-related anxiety or stress [3,23]. In contrast, our study showed poor satisfaction among medical students regarding online teaching methods, especially in terms of acquired clinical skills that require direct observation of their performance by tutors. Similarly, an institute in India that implemented a novel online classroom platform found that 50.9% of the respondents preferred physical classes over online education, despite appreciating the experience [11]. A systematic review and meta-analysis by the Digital Health Education Collaboration in Singapore evaluated the effectiveness of digital education in developing medical students’ communication skills and found low-quality evidence suggesting that digital education is as effective as traditional training [24]. Furthermore, a quantitative cross-sectional study in India, covering 32 medical colleges, evaluated online teaching during the COVID-19 lockdown. Most students (94.4%) found it challenging to study without direct patient exposure [25]. Another study conducted at SRM Medical College Hospital and Research Centre in Trichy, India, assessed medical students’ preferences and perceptions of e-learning and found that the majority of participants (60.8%) preferred classroom teaching [19]. This contrasts with previous literature that reported a positive impact of online medical education during the pandemic [3,20]. We hypothesize that this discrepancy may be attributed to the unique challenges faced by medical students in Jordan, such as limited access to technology and internet connectivity issues, hindering their ability to fully engage with online learning resources. The difficulty of replicating the hands-on clinical experience during online learning may have also contributed to the dissatisfaction of participants.

In this study, most 6^th^-year medical students reported poor communication and clinical skills gained during online education, leading to a lack of confidence in applying their clinical skills. Similarly, in a study conducted at King’s College London to assess medical students’ views on returning to a clinical setting after months of online learning due to the COVID-19 pandemic, the participants reported reduced communication skills and weakened patient–doctor relationships [17].

In our study, 171 participants were 6^th^-year medical students, and 218 were in their 4^th^ and 5^th^ years. We emphasized the perspectives of 6^th^-year medical students compared to those in their 4^th^ and 5^th^ years, because these perspectives could inform decision-makers when considering early graduation in case the pandemic situation worsens in Jordan, a strategy previously implemented in some US and UK medical schools to address the challenges posed by the pandemic [8,26]. Although all study participants agreed that traditional teaching is superior for acquiring new clinical skills, 6^th^-year medical students showed a significantly stronger preference for traditional teaching over online methods compared to 4^th^- and 5^th^ -year students (*P* = 0.003). When asked about their confidence in practicing the clinical skills gained through online learning, there was no significant difference between 6^th^-year students (57.2%) and 4^th^ and 5^th^-year students (49.5%); both groups felt less confident about their clinical skills.

Comparing responses from students at the University of Jordan with those from universities outside Amman revealed no significant differences in terms of tools availability and internet connection problems (*P* = 0.44). This may be attributed to the universities’ locations in vital areas across the country.

### Practical implications

The findings of this study have several practical implications for educators and administrators involved in medical education in Jordan and similar contexts. The most important is the widespread dissatisfaction among medical students with online teaching methods, especially regarding the acquisition of clinical skills, highlighting the need for targeted interventions to enhance the effectiveness of distance e-learning initiatives. Educators should prioritize the development and implementation of innovative teaching strategies that facilitate the acquisition of clinical skills in a virtual environment to ensure that students receive comprehensive training despite the limitations imposed by the online format. Furthermore, efforts should be made to improve the availability of technical resources, especially in rural areas, to mitigate disparities in educational opportunities.

### Study limitations

The major limitation of our study is the inability to measure educational outcomes related to online education and to compare them to traditional education. Further studies are required to address educational outcomes and the perceptions and opinions of faculty staff towards online education.

## CONCLUSION

During the COVID-19 pandemic, distance e-learning became the primary method for continuing medical education. In Jordan, where online teaching was not well-established before the pandemic, this shift presented significant challenges. In this study, medical students expressed dissatisfaction with the clinical skills they acquired through online learning, raising concerns about their confidence in practicing these skills in the future. Technical and infrastructural problems were major barriers to the effective implementation of distance e-learning. Understanding these barriers is crucial for the successful integration of online medical education in Jordan.

## Data Availability

Further data is available from the corresponding author upon reasonable request.
